# Evaluating an online-delivered resistance exercise intervention for racially diverse breast cancer survivors using the RE-AIM framework

**DOI:** 10.1093/tbm/ibag019

**Published:** 2026-04-16

**Authors:** Isabel L Wakefield, Océane Streubel, Adana A. M. Llanos, Shawna V Hudson, Kathryn Schmitz, Siobhan M Phillips, Sharon L Manne, N Lynn Henry, Ken Resnicow, Angela J Fong

**Affiliations:** School of Kinesiology, University of Michigan, Ann Arbor, MI, United States; School of Kinesiology, University of Michigan, Ann Arbor, MI, United States; Department of Epidemiology, Mailman School of Public Health, Columbia University Irving Medical Center, New York, NY, United States; Herbert Irving Comprehensive Cancer Center, Columbia University Irving Medical Center, New York, NY, United States; Institute for Health, Health Care Policy and Aging Research, Robert Wood Johnson Medical School, New Brunswick, NJ, United States; Hillman Cancer Center, University of Pittsburgh, Pittsburgh, PA, United States; Northwestern University Feinberg School of Medicine, Chicago, IL, United States; Division of Population Sciences, Rutgers Cancer Institute of New Jersey, New Brunswick, NJ, United States; Department of Internal Medicine, University of Michigan Medical School, Ann Arbor, MI, United States; School of Public Health, University of Minnesota, Minneapolis, MN, United States; School of Kinesiology, University of Michigan, Ann Arbor, MI, United States

**Keywords:** breast cancer survivors, resistance exercise, online intervention, RE-AIM framework, implementation science

## Abstract

**Background:**

Breast cancer survivors (BCS) experience persistent physical and psychological effects after treatment, with racially diverse groups demonstrating lower adherence to cancer-specific resistance exercise guidelines. Online resistance exercise interventions show promise for enhancing accessibility and health outcomes among BCS. However, evidence regarding their feasibility of implementation remains limited. Yet, such information is critical for assessing scalability and implementation in non-laboratory settings.

**Purpose:**

Using the RE-AIM (reach, efficacy, adoption, implementation, and maintenance) framework, this study evaluated the reach, efficacy, adoption, and implementation of a 12-week, supervised, online resistance exercise intervention for racially diverse BCS.

**Methods:**

A mixed methods approach with a pre–post-study design was used. Quantitative outcomes included sociodemographic representativeness (reach), physical function (efficacy), and session fidelity (implementation). Qualitative interviews examined participant experiences, barriers, and facilitators (adoption).

**Results:**

The intervention enrolled a racially diverse BCS sample (*N *= 47; 57.4% White, 23.4% Black, 14.8% Asian) that was mostly representative of the host institution catchment area, though participants had higher education levels. The intervention group demonstrated statistically significant improvements in upper- (*P *= .009) and lower-body physical function (*P *= .003) versus control, only. Adoption facilitators included program convenience, accessibility of online delivery, and trainer support, while barriers were equipment challenges, competing priorities, and cancer-related side effects. Program implementation fidelity was high for core components.

**Conclusions:**

Key factors contributed to the feasibility of implementation of the intervention. Future remote exercise interventions should address equipment needs, individualized support, and tailored recruitment to enhance adoption and facilitate scalability in non-laboratory settings.

**Clinical Trial Information:**

The Clinical Trials Registration #NCT04562233.

## Teaser

A 12-week supervised online strength training program improved physical function among racially diverse breast cancer survivors, demonstrating strong potential for use in real-world settings. Key factors for success included program accessibility, trainer support, and high delivery quality.

Implications

- **Practice:** Exercise practitioners should deliver online, supervised resistance training for breast cancer survivors with built-in equipment support and flexibility to accommodate participants’ schedules and treatment side effects.
- **Policy:** Policymakers should fund and promote accessible, scalable online resistance exercise programs tailored to racially and ethnically diverse cancer survivor populations to advance health equity in survivorship care.
- **Research:** Researchers should refine recruitment methods and intervention strategies to maximize participation, individualize support, and identify scalable features for sustained adoption of remote exercise programs among breast cancer survivors.

## Introduction

In the United States, the 5-year relative survival rate for breast cancer is 91% [1]. Breast cancer survivors (BCS), defined from the time of diagnosis onward [2], who identify as American Indian/Alaskan Native, Black, or Hispanic experience lower survival rates compared to their White counterparts [3]. BCS often report experiencing treatment-related physical effects such as fatigue, pain, reduced physical function, and decreased muscle mass, as well as psychological effects and cognitive impairment, which can diminish health-related quality of life long after treatment has ended [4–7]. Thus, interventions that mitigate physical and psychological effects are needed among American Indian/Alaskan Native, Black, and Hispanic BCSs. Physical activity (PA) is associated with reductions in treatment-related effects and enhanced health-related quality of life [8, 9]. Resistance exercise (i.e. weightlifting or strength training such as bicep curls with weights or bands and push-ups with body weight) offers independent health benefits such as increased muscle mass and improved physical function, which are associated with higher health-related quality of life [10–12].

In recognition of the benefits of resistance exercise, the American College of Sports Medicine recommends that cancer survivors engage in two or more days per week of resistance exercise [8, 13]. However, adherence is low, with only 17.6% of BCSs meeting resistance exercise guidelines [14]. Moreover, recent data from the National Health Interview Survey indicate that only 10.3% of Black BCS meet recommended resistance exercise guidelines regardless of whether aerobic guidelines (150 min of moderate-to-vigorous PA per week) are met compared to 18.9% of their Non-Hispanic White counterparts [14]. As a result, interventions focused on increasing resistance exercise adherence that include racially diverse study populations are needed. Furthermore, a critical gap exists in the transition of these interventions from controlled laboratory to non-laboratory settings. To bridge this gap, research is needed to assess factors that impact intervention implementation. The RE-AIM framework is commonly used to evaluate the feasibility of implementation and public health impact of interventions and consists of five dimensions: Reach, Efficacy, Adoption, Implementation, and Maintenance [15–17]. This assessment is crucial for enabling the broader dissemination and scaling of such interventions to reach target populations.

The Breast cancer Resiliency through Exercise Program (B-REP) was developed to mitigate issues related to lack of accessibility and poor sample representativeness [12, 18, 19]. B-REP was a two-arm randomized controlled trial with participants randomized to either an intervention or minimal contact control (MCC) group and completed assessments at pre- and post-study [13]. The intervention was a 12-week, supervised, online-delivered, once-weekly resistance exercise intervention for racially diverse BCS [13]. The intervention specifically targeted racially diverse BCS in the extended survivorship phase, which is 6-month to 5-year post-diagnosis [20]. This timeframe represents a period where acute treatment effects have stabilized, yet long-term recovery of muscle mass and physical function are important [8, 9]. The pilot study was rated as feasible and acceptable and increased upper- (chest press) and lower-body (deadlift) strength as measured by 10-repetition maximum tests [13]. Although B-REP demonstrated initial promise, factors contributing to its feasibility of implementation are unclear. Evaluating the factors that contribute to the intervention’s feasibility of implementation is essential for identifying those that can be adapted for eventual scale-up [15]. The feasibility (i.e. recruitment and retention) and acceptability (i.e. adherence to the intervention and satisfaction) of B-REP, as well as preliminary findings on its effects on muscular strength, self-reported health-related quality of life, and self-efficacy, have been previously published [13]. In contrast, the aim of the current analysis is to evaluate the public health impact of B-REP using the RE-AIM framework (i.e. there is no duplication) [15]. Specifically, we sought to (i) assess the extent to which a representative sample of the catchment area was enrolled into B-REP (reach); (ii) assess the preliminary efficacy of the intervention for improving physical function and cardiorespiratory fitness (efficacy); (iii) qualitatively explore the individual-level facilitators and barriers impacting adherence (adoption); and (iv) assess the fidelity of intervention delivery (implementation). Since B-REP was a pilot study without a longer post-intervention follow-up assessment, it is not possible to evaluate the maintenance of the intervention.

## Methods

### Study design

We used a mixed-methods approach with a pre–post-study design. A complete description of the B-REP pilot study was published previously [13]. Institutional review board approval was obtained from the original host institution (Rutgers Cancer Institute) to conduct the research (IRB# Pro2021002042) and a data user agreement (23- UFA04108) with the University of Michigan following the senior author’s (A.J.F.) change of institution to analyze the deidentified data.

### Participants and recruitment

Participants were recruited using case ascertainment through the electronic medical record database, physician referral, and in-clinic using flyers at the host institution. Interested individuals completed an eligibility screening questionnaire in-person, over the phone, or online. Research staff confirmed eligibility and completed the informed consent process in-person, over the phone, or online using an electronic form. Women were eligible to participate if they: (i) were 18 years of age or older, (ii) had a physician-confirmed diagnosis of Stages 0 to IIIa breast cancer within the past 5 years, (iii) did not have a concurrent cancer diagnosis, (iv) had completed primary treatment at least six months prior to the study, (v) were not currently meeting cancer-specific resistance exercise guidelines with no restrictions based on aerobic activity, (vi) were deemed safe to exercise based on the Get Active Questionnaire [21], (vii) could read and understand English, and (viii) had reliable access to an internet-connected device with a video camera. Women were excluded if they: (i) had a diagnosis of metastatic disease, (ii) planned to undergo elective surgery during the intervention period, or (iii) intended to move out of the United States during the study period. Recruitment occurred between October 2020 and February 2022.

### B-REP intervention

Briefly, B-REP was a 12-week, supervised, online-delivered resistance exercise intervention among racially diverse BCS [13]. Participants were randomly assigned to either the intervention or MCC groups. The intervention group attended weekly 30- to 45-min one-on-one supervised Zoom sessions. Given the feasibility nature of the study, exercise sessions were only offered weekly [13]. The MCC group received individualized exercise booklets with written and photographic instructions. All participants were given an individualized resistance training plan based on baseline testing.

### Measures and procedures

Assessments occurred at baseline (Week 0) and post-intervention (Week 12). Data sources for reach, efficacy, adoption, and implementation dimensions included data extracted from the electronic medical record, online questionnaires, in-person physical function assessments, study documents, one-on-one interviews, and publicly available databases. A summary of the measures as they are aligned with the RE-AIM dimension is in Table 1 and described in greater detail below.

**Table 1:** A summary of the data sources and assessments for RE-AIM dimensions.

Dimension	Operationalization	Data sources	Assessment timepoint(s)
Reach	Sociodemographic representativeness of participants compared to the host institution catchment area.	Online questionnaire State census database	Baseline
Efficacy	Impact of B-REP on physical function outcomes.	In-person physical function assessment	Baseline and post-intervention
Adoption	Individual-level factors impacting engagement in B-REP.	One-on-one interviews	Post-intervention
Implementation	The intervention was delivered as intended.	Fidelity checklist	Throughout intervention (Weeks 1–12)

The maintenance dimension was not assessed in the current study.

#### Reach

Sociodemographic variables were self-reported using an online questionnaire and included age (years), race [White, Black or African American, Asian or Asian American, or Other (including American Indian or Alaska Native, Native Hawaiian or other Pacific Islander, and multiracial)], and Hispanic ethnicity (yes or no), current marital status (married, divorced, separated, or single/never married), and employment status (employed/self-employed, unemployed/homemaker/student/disabled, or retired). Data extracted from the electronic medical record included cancer diagnosis, staging, and information on treatments received. National-level information on median age at breast cancer diagnosis was abstracted from the Surveillance, Epidemiology, and End Results database because an accurate representation of median age in New Jersey state could not be identified [22]. State-level racial and ethnic distribution for breast cancer cases were abstracted from the New Jersey State Cancer Registry [23]. State-level data on marital status, education, household income, and employment status were abstracted from the United States Census Bureau [24].

#### Efficacy

Physical function including upper- and lower-body physical function and cardiorespiratory fitness were assessed at baseline and post-intervention using a subset of the Senior Fitness Test battery [25]. Due to staffing constraints, the exercise trainer also assessed outcomes.

Upper body physical function was assessed using the 30-s arm curl test. Participants sat in a chair with no armrests and repeatedly curled a 5 lb (2.27 kg) with their dominant arm as many times as possible for 30 s. Each curl began with the palm facing the body and rotated to face the shoulder at the top. Feet remained flat on the floor and their back remained as straight as possible. After two practice trials, the number of repetitions was recorded.

Lower body physical function was assessed using the 30-s chair sit-to-stand. Participants sat in a chair with no armrests that was placed against a wall and performed as many full stands as possible in 30 s. Arms were crossed across their chest, feet remained flat on the floor, and back remained as straight as possible. After two practice trials, the number of repetitions was recorded.

Cardiorespiratory fitness was assessed using the 6-min walk test. Participants walk a 100-ft (30 m) course marked with orange traffic cones at a self-selected pace for 6 min. Participants were permitted to stop and rest as needed, but the timer was not stopped. Total distance walked was recorded in meters.

#### Adoption

At post-intervention, one-on-one, semi-structured interviews were conducted via video conferencing software (Zoom) by a research staff member who was blinded to the study purpose. The rationale for a qualitive exploration was to assess factors that impacted adoption of the intervention and inform future scalability. To this end, a subset of intervention participants (*n *= 4) was invited to participate in the interviews. Participants were purposively selected based on their session attendance—two with high attendance (attended all sessions) and two with lower attendance (attended 58%–80% of sessions)—and their self-identified race, with all four identifying as Black. Black participants were invited because this racial group enrolled at a higher rate compared to other racial groups combined (64.7% vs. 30.7% of those eligible) without a tailored recruitment strategy. A semi-structured interview guide was developed guided by Social Cognitive Theory [26] and previous research [27]. The questions focused on perceived individual-level factors impacting adoption of B-REP.

#### Implementation

All supervised exercise sessions were recorded (*N *= 312). A random subset of videos representing 5% (*n *= 16) of the recordings were selected for coding. Using a fidelity checklist, a research staff member who was blinded to the purpose of the study, completed the checklist after being trained by the senior author (A.J.F). The fidelity checklist was developed based on the Component Level Implementation Fidelity Rating System [28], which emphasizes assessing manual adherence, exposure and dosage, delivery quality, participant responsiveness, and intervention differentiation. Following this framework, the checklist included observable components to ensure consistent delivery and evaluation of key components across sessions. Checklist items included: (i) a review of the previous session, (ii) a warm up lasting at least 5 min, (iii) completion of the full workout of 8 exercises, (iv) appropriate cueing and modifications, (v) 30 s to 1 min breaks between exercises, (vi) a 5–10 min cooldown, (vii) reminder of next session, (viii) time for participant questions, (ix) a reminder to increase load or resistance during designated progression Weeks 4 and 8, and (x) a reminder of next assessment time point at Week 11 or 12.

### Data analysis

Quantitative data for the reach, efficacy, and implementation dimensions were summarized using descriptive statistics including means, standard deviations, and percentages. For reach, a visual comparison was used to compare the medians for age of B-REP participants compared to the national age at breast cancer diagnosis [22]. Pearson chi-square tests were conducted for all remaining sociodemographic variables followed by *post hoc* standardized residuals to compare significant comparisons to determine over- and underrepresentation. For efficacy, a 2 × 2 (group by time) factorial analysis of variance (ANOVA) was used to compare the effects of randomization and time on physical function outcomes. Partial eta-squared ($\eta_{p}^{2})$ effect sizes were reported, where a small effect is 0.01, a medium effect is 0.06, and a large effect is 0.14 [29]. For implementation, percentages were used to summarize the data and visually compared to the *a priori* benchmark of > 80%. Statistical tests were two-sided and significant at *P* < .05. Analyses were conducted using the Race for Your Life package in R (version 4.4.1).

Qualitative data were analyzed deductively using content analysis [30, 31]. We conservatively anticipated *n *= 5 participants to reach thematic saturation, which is defined as when no new themes or patterns develop [32, 33]. However, after a preliminary analysis of the four interviews, it was determined that data saturation had been reached and a fifth interview was not needed. The interviews were transcribed verbatim. Two authors (O.S. and I.L.W.) independently reviewed the transcripts for familiarity and coded the transcripts. After initial coding, the two authors compared their codes and condensed and deleted irrelevant codes to ensure consistency of the codebook. Discrepancies were settled via consensus with a third research staff member who was not part of the original research or the current study. The updated codebook was used to re-code the interviews. Analyses were conducted using NVivo 15 (Lumivero LLC, Denver, CO).

## Results

### Participants

A total of 47 participants enrolled in B-REP, and the final analytical sample is *N = *39 after attrition (*n *= 26 in the intervention and *n *= 13 in MMC conditions). Eight participants (*n *= 2 intervention and *n *= 6) were lost at follow-up. Overall, participants were racially diverse (57.4% White, 23.4% Black, and 14.8% Asian), were married (69.6%), had earned a bachelor’s degree or higher (74.0%), employed (56.5%), had a household income of $75 000 or higher (57.8%), primarily diagnosed with Stage I breast cancer (58.1%), and had completed treatment *M *= 35.4 (SD = 18.2) months prior to study start and 63.0% were on hormonal therapy. Refer to Table 2 and the previous publication for details [13].

**Table 2:** Participant sociodemographic characteristics statistically compared to national- and state-level data.

Characteristic	B-REP *n* (%)	Catchment area *n* (%)	χ²	*z*	*P*
Race			2.97		.255^a^
White	27 (57.4)	6452 (68.0)^b^			
Black/African American	11 (23.4)	1177 (12.41)^b^			
Asian/Asian American	7 (14.8)	715 (7.54)^b^			
Other	2 (4.3)	n/a			
Ethnicity			0.67		.264^a^
Hispanic	3 (6.4)	1060 (11.2)^b^			
Non-Hispanic	44 (93.6)	8426 (88.8)^b^			
Marital status			3.13		.081^a^
Married	32 (69.6)	1 828 874 (46.9)^c^			
Not married	14 (30.4)	1 731 386 (44.4)^c^			
Household income			6.14		.185^a^
$10 000–$24 999	6 (13.3)	277841 (7.90)^c^			
$25 000–$49 999	1 (2.2)	485342 (13.80)^c^			
$50 000–$74 999	5 (11.1)	481825 (13.70)^c^			
$75 000–$99 999	9 (20.0)	407969 (11.60)^c^			
$100 000 or more	17 (37.8)	1749904 (49.50)^c^			
Prefer not to answer	7 (15.6)	n/a			
Education			10.97		.009^a^
High school	2 (4.3)	826201 (24.8)^c^		−2.75^d^	
Post-secondary	1 (2.2)	n/a			
Some college	3 (6.5)	495455 (14.8)^c^		−1.20^d^	
Associate’s degree	6 (13.0)	426669 (7.40)^c^		1.06^d^	
Bachelor’s degree	20 (43.5)	876000 (26.2)^c^		2.35^d^	
Graduate degree	13 (28.3)	n/a			
Other	1 (2.2)	n/a			
Employment			2.83		.121^a^
Employed	32 (69.6)	2 216 469 (57.7)^c^			
Unemployed	11 (23.9)	1 622 951 (42.3)^c^			
Prefer not to answer	2 (4.3)	n/a			
Other	1 (2.2)	n/a			

a Chi-square test for independence.

b Sociodemographic data from the New Jersey State Cancer Registry.

c Sociodemographic data from the United States Census Bureau.

d Standardized residuals, values ≥ |1.96| indicate significant overrepresentation (positive) or underrepresentation (negative).

### Reach

Based on a visual comparison, participants in B-REP were younger (median = 55 years) than the national median age at breast cancer diagnosis (median = 63 years). A national median was used because accurate state-level data could not be found. Chi-squared analyses indicated no statistically significant differences between the B-REP participants and the 2022 New Jersey census data or New Jersey State Cancer Registry in the distribution of race (*χ*^2^ = 2.97, *P *= .26), ethnicity (*χ*^2^ = 0.67, *P *= .26), household income (*χ*^2^ = 6.14, *P *= .19), employment status (*χ*^2^ = 2.83, *P *= .12), and marital status (*χ*^2^ = 2.63, *P *= .09). However, statistically significant differences were observed for education level (χ^2^ = 10.70, *P *= .009). Follow-up standardized residuals revealed that individuals holding a bachelor’s degree were overrepresented among B-REP participants (*z* = 2.35), while individuals with a high school degree were underrepresented (*z* = −2.75). The chi-square tests are summarized in Table 2.

### Efficacy

Descriptive statistics, including group means and standard deviations, are presented in Table 3. The 2 × 2 ANOVAs indicated a statistically significant main effect of study group and time for upper body physical function (arm curl test), *F*(1, 38) = 5.40, *P *< .001, $\eta_{p}^{2}$ = 0.166, and lower body physical function (chair sit-to-stand test), *F*(1, 38) = 10.26, *P *= .003, $\eta_{p}^{2}$ = 0.213. No significant differences were observed for cardiovascular fitness (6-min walk test), *F*(1, 38) = 2.98, *P *= .092, $\eta_{p}^{2}$ = 0.073.

**Table 3:** Comparing physical function outcomes between B-REP and MCC groups (*N* = 39).

	Intervention group (*n *= 26)		MCC group (*n *= 13)		df	*F*	$\eta_{p}^{2}$	*P*
Physical function test outcomes	*M* (SD) Baseline	*M* (SD) Week 12	*M* (SD) Baseline	*M* (SD) Week 12				
Upper body physical function: arm curl (repetitions)	19.07 (3.54)	22.74 (4.56)	18.15 (5.18)	18.85 (5.74)	1, 38	5.40	0.166	.009
Lower body physical function: chair sit-to-stand (repetitions)	12.81 (3.03)	16.89 (5.02)	12.00 (2.86)	12.92 (4.25)	1, 38	10.26	0.213	.003
Cardiovascular fitness: 6-min walk (m)	434.85 (87.69)	479.77 (87.88)	444.66 (80.14)	460.72 (94.61)	1, 38	2.98	0.073	.092

### Adoption

Across all interviews, three main themes from *N *= 4 participants’ experiences in B-REP were identified including facilitators of adoption (37 total counts), barriers to adoption (eight total counts), and feedback to facilitate future adoption (23 total counts). Facilitators of adoption also included three subthemes: (i) personal motivation, (ii) intervention components, and (iii) influence of the exercise trainer. Barriers to adoption also had three subthemes: (i) intervention equipment, (ii) competing priorities, and (iii) treatment-related side effects. Themes with exemplary quotes and frequency counts are summarized in Table 4.

**Table 4:** Summary of adoption findings from interviews with purposively sample participants (*n* = 4).

Adoption themes (frequency)	Adoption sub-themes (frequency)	Exemplary quotes
Facilitators (total count = 37)	Intervention components (counts = 21)	“[Zoom] made it even better because it took out having to like having to drive in and then drive home. So that would take up even more time, so doing it on Zoom was to me like being there in person. I thought that was really good.” -P57
	Influence of the exercise trainer (counts = 10)	“A personal trainer who’s had the same walk that I’ve had [referring to a cancer diagnosis] that can relate to me in more than just—or with me in more than just physical.” -P40
	Personal motivation (counts = 6)	“I think it was just, like I said, the fact that I knew I needed to do something, I needed to do some type of strength and conditioning.” -P65
Barriers (total count = 8)	Intervention equipment (counts = 2)	“I mean, if I can think of anything probably just the bands. They always intimidated me, they still intimidate me because I think they’re gonna snap.” -P40
	Competing priorities (counts = 4)	“And I had a lot going on. You know and having to help with two six-year-old grandkids and you know, with my daughter, and trying to work, and being in COVID. It was just so much. Very very overwhelming.” -P57
	Treatment-related side effects (counts = 2)	“[….] I still have issues from all of my treatments that I was going through, like the feet and the tingling in the hands and things like that. So some of those things are a little bit harder for—some of those things make it a little bit harder for me to do the exercise—to exercise, period. And then I just give up because, you know, either I’m in pain that day.” -P65
Feedback to facilitate adoption (total count = 23)		“You know, the dumbbells would be good only because, you know, that would be good for my arm training. You know?” -P65 “Something maybe something like a chart for healthy eating or you know.” -P57 “[…] maybe instead of having it once a week, maybe twice a week?” -P68

Commonly reported facilitators of adoption were intervention-related components such as the one-on-one supervised sessions, easy-to-use equipment, program convenience and accessibility of Zoom. Additionally, participants discussed the strong positive influence of the exercise trainer and the role of personal motivation in supporting their engagement.

Barriers to adoption primarily reflected practical and personal challenges. Competing priorities, such as work obligations and caregiving responsibilities, were frequently cited as obstacles to consistent participation. Treatment-related side effects such as neuropathy in their hands or feet, also hindered some participant’s ability to engage in the intervention. Additionally, some participants expressed concerns related to the intervention equipment, noting discomfort in using resistance bands and accelerometers.

Factors to consider for future iterations of B-REP included adding nutritional information, increasing program frequency and duration, and the addition of other types of resistance exercise equipment, such as dumbbells.

### Implementation

Fidelity was consistently above the *a priori* 80% benchmark across core components of the intervention, with 100% fidelity to warm-ups, full workouts, cueing and modifications, and cool-downs. Areas that did not meet the benchmark included review of the previous session (43.8%), time allotted for participant questions (75.0%), and reminders to increase load or resistance when appropriate (25.0%). The checklist item “reminder for the next assessment time point at Week 11 or 12 only” was removed because none of the randomly selected videos were from Week 11 or 12 of the intervention. A summary of all fidelity checklist items is in Table 5.

**Table 5:** Summary of B-REP intervention implementation fidelity checklist.

Fidelity checklist item	Observed (%)^a^
Review of previous session	43.8
Warm up > 5 min	100.0
Full workout (all eight exercises)	100.0
Cueing and modifications	100.0
Breaks between major exercises (30 s to 1 min)	81.3
Cool down (5–10 min)	100.0
Reminder of next session	87.5
Time for questions	75.0
Reminder of increase in load or resistance (Weeks 4 and 8 only)	25.0

a Compared to *a priori* benchmark of 80%. The checklist item “reminder for the next assessment time point at Week 11 or 12 only” was removed because none of the randomly selected videos were from Week 11 or 12 of the intervention.

## Discussion

The current analysis aimed to use the RE-AIM framework to evaluate an online-delivered resistance exercise intervention among racially diverse BCSs called B-REP [15]. The key findings for each dimension are discussed below.

### Reach

B-REP participants were representative of the host institution’s catchment area in terms of race, ethnicity, household income, employment status, and marital status based on the non-significant Chi-square tests. In contrast, participants had statistically significantly higher education compared to the catchment area. Because recruitment was conducted using electronic medical records, it is possible that the study sample reflected the diversity of the county where the host institution is located, where 55.8% of residents self-identify as races other than White and 47.5% hold a bachelor’s degree, rather than that of the catchment area overall [24]. Electronic medical record recruitment has been shown to be effective for clinical trial enrollment [34, 35]; however, individuals outside of the healthcare system are likely not reached [36]. To enhance representativeness in future interventions, recruitment strategies should implement community-based initiatives that offer multiple contact methods, use diverse outreach channels, foster community engagement and trust, and highlight community-wide benefits of participating in the study [37].

### Efficacy

Our findings that B-REP demonstrated preliminary efficacy in improving physical function for upper- and lower-body physical function for the intervention group compared to the MCC group align with existing evidence showing that resistance exercise interventions improve physical function among BCSs following treatment [38–40]. Although the once-weekly supervised dose of B-REP did not meet cancer-specific guidelines because of the pilot study design, the improvements in physical function suggest that a lower dose may still yield health benefits [8]. These findings are encouraging; however, further assessment in a fully powered trial is needed to confirm effectiveness. Similarly, there were no changes to cardiorespiratory fitness and further research is needed to examine the effect of resistance exercise on cardiovascular health outcomes [41].

### Adoption

Participants stated that the remote delivery of the intervention was a key facilitator. While the finding is consistent with prior research, a potential unintended outcome is that individuals’ digital access issues or low comfort with videoconferencing may have been excluded. Moreover, barriers included competing responsibilities and treatment-related side effects, which is consistent with previous research [42]. Future research is encouraged to overcome access barriers by providing mobile hotspots, loaning devices, and providing technology onboarding tutorials to lower the barrier to entry for those with digital access issues [43]. Oncology-trained exercise professionals should tailor protocols to mitigate treatment-related side effects and address psychosocial barriers and facilitators. To increase adoption, future research should assess setting-level influences, such as physical environments, clinic workflows, and staff engagement may facilitate the likelihood of uptake and inform sustainability of the intervention [15].

### Implementation

B-REP demonstrated high fidelity for core intervention components. But fidelity was lower for interactive elements such as reviewing previous sessions and allowing time for questions. The high fidelity for core components suggests that the study-specific training and exercise trainer education were effective, which is notable since complex interventions often face greater risks of misinterpretation or inconsistent delivery [44]. However, the lower fidelity for interactive components may reflect the tension inherent to synchronous remote intervention delivery, where time spent on technical setup or connectivity issues can compress time available for soft-skills engagement. Although these findings are encouraging, further assessment in a fully powered trial is needed to confirm effectiveness. Additionally, there were no changes to cardiorespiratory fitness and further research is needed to examine the independent effect of resistance exercise compared to aerobic exercise on cardiovascular health outcomes.

### Limitations

The study is not without its limitations. One key limitation is the absence of follow-up assessments beyond the post-intervention time period, which did not allow for evaluation of the maintenance dimension of RE-AIM [15]. Understanding the maintenance effects of the intervention warrants further investigation to better understand the impact of the intervention. While B-REP demonstrated preliminary efficacy for improving upper- and lower-body physical function, these results should be interpreted with caution due to the small sample size, attrition, potential bias introduced by the exercise trainer assessing outcomes, and because the study was primarily designed to assess feasibility rather than efficacy [13].

## Conclusions

Overall, the high implementation fidelity likely contributed to the demonstrated preliminary efficacy for improving physical function, suggesting that the remote delivery is suitable for a resistance exercise intervention. However, while the delivery mode was engaging to the racially diverse participants, the overrepresentation of highly educated participants suggests that digital access and/or literacy may be potential barriers. To maximize public health impact, future research should include technology onboarding to ensure equitable access across socioeconomic strata.

## Data Availability

De-identified data from this study will be made available (as allowable according to institutional IRB standards) by emailing the corresponding author.

## Welfare of animals

This article does not contain any studies with animals performed by any of the authors.
